# The impact of intravertebral cleft on cement leakage in percutaneous vertebroplasty for osteoporotic vertebral compression fractures: a case-control study

**DOI:** 10.1186/s12891-021-04685-9

**Published:** 2021-09-18

**Authors:** Benqiang Tang, Songjie Xu, Xueming Chen, Libin Cui, Yanhui Wang, Xin Yan, Yadong Liu

**Affiliations:** Department of Spinal Surgery, Beijing Luhe Hospital, Capital Medical University, No.82, Xinhua South Road, Touzhou District, Beijing, 101149 China

**Keywords:** Intravertebral cleft, Cement leakage, Percutaneous vertebroplasty, Osteoporotic vertebral compression fracture

## Abstract

**Background:**

The impact of intravertebral cleft (IVC) on cement leakage in percutaneous vertebroplasty (PVP) for osteoporotic vertebral compression fractures (OVCFs) has been discussed. However, the results were conflicting, as the study population and cement leakage classification were heterogeneous. The aim of the study was to evaluate the impact of IVC on the incidence of leakage through vein, leakage through cortex as well as general leakage in PVP for OVCFs.

**Methods:**

All patients with OVCFs who underwent PVP between January 2016 and June 2019 at our institution were retrospectively reviewed. Patients were eligible for this case-control study if they were diagnosed as single level fracture in spine. After inclusive and exclusive criteria were met, a total of 139 patients with IVC were enrolled as the study group. Non-IVC controls were matched in a 1:1 ratio in age (within 3 years), sex and fracture severity with patients in study group. Cement leakage were classified into four types [type B (through basivertebral vein), type S (through segmental vein), type-C (through a cortical defect), and type D (intradiscal leakage)], furtherly into two types [venous type (type-B or/and type S) and cortical type (type-C or/and type-D)]. A general leakage rate and a specific leakage rate per each type were compared between both groups.

**Results:**

Each group included 139 patients. Groups were homogenous for age, sex, fracture severity, fracture location, fracture type, cement volume, puncture approach and property of cement. Compared with control group, IVC group had a significantly lower rate of type-B (20.9% vs. 31.7%, *P* = 0.041), type-S (24.5% vs. 52.5%, *P* = 0.000), and venous type leakage (37.4% vs. 67.6%, *P* = 0.000), a significantly higher rate of type-C (25.9% vs. 12.2%, *P* = 0.004), type-D (16.5% vs. 6.5%, *P* = 0.009), and cortical type leakage (40.3% vs. 16.5%, *P* = 0.000), no significant difference on the rate of general leakage (67.6% vs. 76.3%, *P* = 0.109).

**Conclusion:**

IVC decreased the risk of cement leakage through vein and increased the risk of cement leakage through cortex. However, it had no significant effect on the occurrence of general leakage.

## Background

The intravertebral cleft (IVC) was generally considered to be a sign of avascular osteonecrosis of the vertebral body [1–3]. Most IVCs occur at the thoracolumbar junction, where flexion and extension of the spine is most dynamic [3, 4]. As a result, this may predispose to the occurrence of nonunion or pseudarthrosis in fractured vertebrae [3, 4]. The rate of IVC in osteoporotic vertebral compression fractures (OVCFs) varied from 13.8 to 42.4% in recent series [5–11]. Regardless of the presence of IVC, percutaneous vertebroplasty (PVP) had provided satisfactory clinical and radiological outcomes [5, 12–16]. Cement leakage was the most common complication [12–17].

The impact of IVC on cement leakage in PVP for OVCFs has been discussed [5–9, 13–15, 18–21]. In previous series, the results of its impact on specific type leakage were conflicting, as the study population and cement leakage classification were inhomogeneous [5–9, 13–15, 18, 19]. In two meta-analysis, the results of its impact on general leakage should be carefully weighted as their eligible studies included were heterogeneous in procedure (i.e., vertebroplasty or kyphoplasty) [20, 21]. Hence, the impact of IVC on cement leakage in PVP for OVCFs may remain unclear.

To our knowledge, this is the first case-control study focusing on this topic. The purpose of the study is to compare the incidence of leakage through vein, leakage through cortex, as well as general leakage in PVP for OVCFs with and without IVC. In addition, the implication of the results will be discussed.

## Methods

### Patient selection

Following institutional review board approval, a retrospective case-control study was conducted involving all the patients with OVCFs who underwent PVP at the authors’ hospital between January 2016 and June 2019. OVCF was defined as a fragility fracture secondary to a low energy mechanism of injury, or a concomitant bone mineral density (BMD) T-score of less than or equal to 2.5 at the spine. All vertebra fracture diagnosis was made after clinical and radiological assessment of the spine. Of note, we limited subjects in the current study to patients who was diagnosed as single level fracture in spine.

As randomized controlled trials indicated that vertebroplasty was associated with greater pain relief and improved functional outcomes for acute, subacute or chronic OVCFs compared with conservative treatments [22–25], in our institution early vertebroplasty or kyphoplasty was recommended to OVCF patients once diagnosed. Indications to vertebroplasty or kyphoplasty may vary among centers. In our center, case of mild or moderate fractures were usually indicated to vertebroplasty, while cases of severe fractures had been considered as candidates for percutaneous kyphoplasty or spinal reconstructive surgery.

**Fig. 1:**
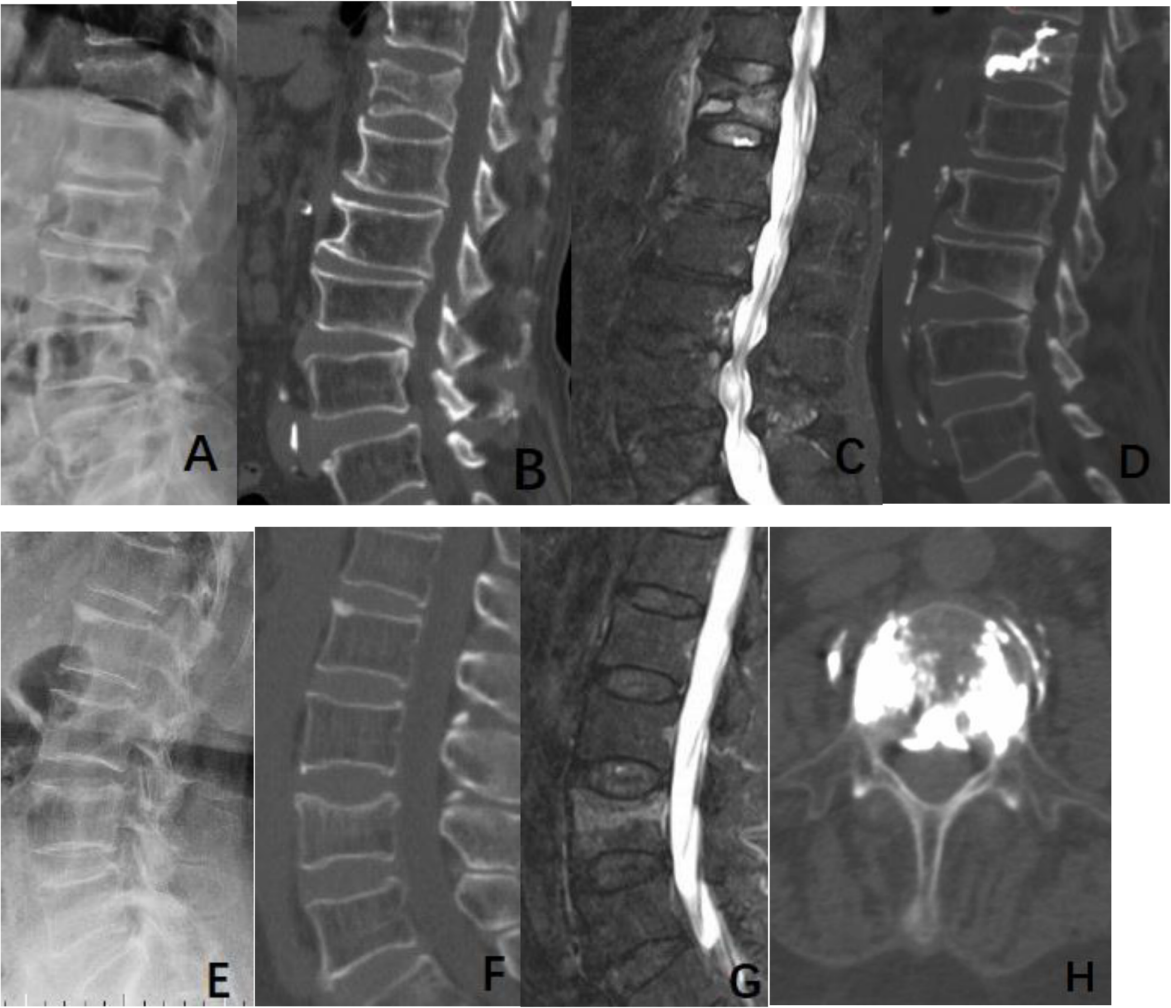
Case 1 (**a**, **b**, **c**, **d**) was an 88-year-old male patient. A moderate and biconcave type fracture in T12 was shown on lateral radiograph (**a**). An IVC in T12 was confirmed on sagittal CT reconstruction (**b**) and MRI (**c**). An area similar to fluid in this IVC was demonstrated on sagittal MRI (**c**). Leakage was detected through the upper endplate of T12 vertebra on postoperative CT (**d**). Case 2 (**e**, **f**, **g**, **h**) was a 71-year-old female patient. There was evidence of a mild and wedge type fracture in L4 on lateral radiograph (**e**). A L4 fracture with absence of the IVC was confirmed on sagittal CT reconstruction (**f**) and MRI (**g**). Leakage was detected into spinal canal and segmental vein on postoperative CT (**h**)

Clinical and surgical data obtained from medical files included age, sex, duration of fracture, fracture location, cement volume. Radiological data included fracture severity, fracture type, and IVC. Fracture severity was classified according to the percentage of vertebral body collapse as mild (20–25%), moderate (26–40%), and severe (>40%) on plain lateral radiographs of the spine, using the classification of Genant et al. [26] (Fig. 1). Fracture type was denominated according to the semiquantitative classification of Genent et al. as either wedge, biconcave, or crush [26] (Fig. 1). IVC was defined as an intravertebral transverse, linear or cystic radiolucent shadow on preoperative CT, as a hypointense area similar to air on T1-weighted MRI sequences and on T2-weighted MRI sequences, or as a hyperintense area similar to cerebrospinal fluid on MRI STIR sequences [12] (Fig. 1). The radiological data were evaluated independently by two authors (B.Q.T. and L.B.C.), with discrepancies resolved by a third author (X.M.C).

Totally, data of potential subjects were available for 1089 patients, including 267 (24.5%) patients with IVC and 822 (75.5%) patients without IVC. To control for possible confounders, the rigorous inclusion and exclusion criteria were used. The inclusion criteria were (1) aged at 60 years or older; (2) fracture location in thoracolumbar junction; (3) mild or moderate fracture. The exclusion criteria were previous spinal surgery. After the inclusion and exclusion criteria were met, 539 patients remained, including 139 patients with IVC and 400 patients without IVC. All the 139 patients with IVC were enrolled as the study group. Controls were randomly selected that were matched in a 1:1 ratio in age (within 3 years), sex and fracture severity.

### Technical note

A routine bilateral transpedicular approach was used to perform PVP. After the needle was inserted into an IVC, polymethylmethacrylate (PMMA) bone cement was injected to fill this cavity. The injection was stopped when the entire cleft was filled. When there was absence of cleft in a fractured vertebra, the needle was positioned to the optimal position, that was, fractured cancellous bone. The injection was stopped when this optimal position was filled or progressive symmetrical satisfactory filling of the vertebral body was noted on intraoperative live fluoroscopy images. In addition, injection was stopped when leakage was detected during the procedure for any fractured vertebra. All the PMMA bone cement injected was low-viscosity in properties. All the injection was conducted during the “toothpaste-like” phase.

### Evaluation of cement leakage

**Fig. 2:**
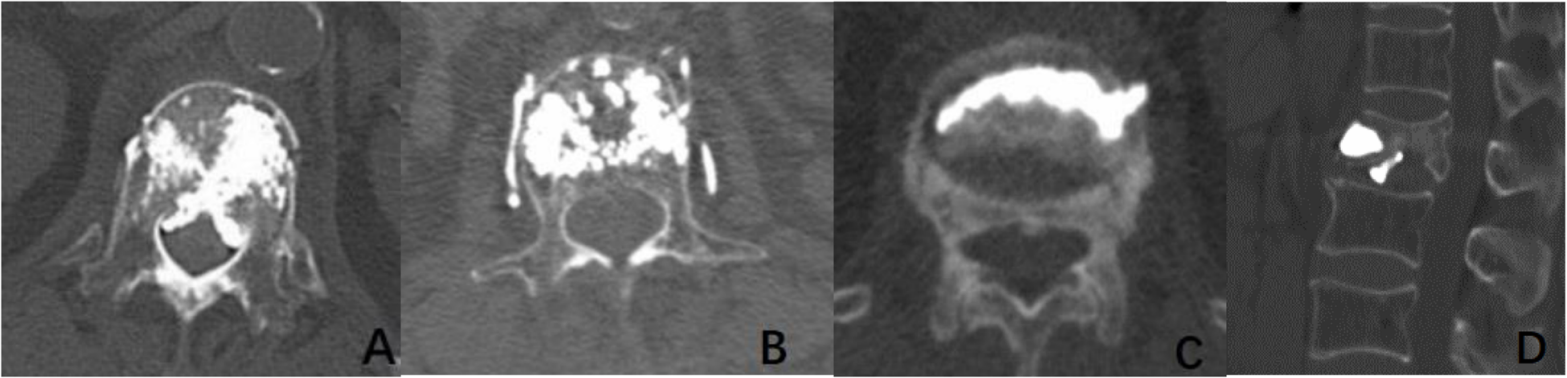
Types of leakage. **a**. Type B (through the basivertebral vein). **b**. Type S (through the segmental vein). **c**. Type C (through a cortical defect). **d**. Type D (intradiscal leakage). The venous type: **a**, **b**. The cortical type: **c**, **d**

Cement leakage were assessed on the routine postoperative computed tomography (CT) scanning of all treated levels. Cement leakage were classified into 4 types: through the basivertebral vein (type B), through the segmental vein (type S), through a cortical defect (type-C), and intradiscal leakage (type D) [27, 28] (Fig. 2). Furtherly, any cement leakage was stratified into two types: the venous type, when leakage was detected in the basivertebral vein (type-B) or/ and segmental vertebral vein (type-S), and the cortical type, when leakage was detected through cortical disruption at the wall of the vertebral body (type-C) or/and at the endplates (type-D) [29, 30] (Fig. 2). Cement leakage were also evaluated independently by two authors (B.Q.T. and L.B.C.), with discrepancies resolved by the third author (X.M.C).

### Statistical analysis

Descriptive statistics were presented as frequencies (percentages) for categorical variables and as means with standard deviations for continuous variables. Chi-square tests were used for the evaluation of categorical variables. Independent-samples *t* tests were used to analyze continuous variables. All statistical tests were 2-sided and *P* values less than 0.05 were considered to be statistically significant.

## Results

Each group included 139 patients. Baseline characteristics of the two groups were summarized in Table 1. No significant difference between two groups were seen in age (*P* = 0.957), sex (*P* = 1.000), fracture severity (*P* = 1.000), fracture type (*P* = 0.059), and cement volume (*P* = 0.329). In addition, fracture location (thoracolumbar junction), puncture approach (bilateral transpedicular puncture) and property of cement (low-viscosity) were the same in two groups, respectively. However, duration of fracture (days) was significantly greater in the IVC group compared with the control group (15.4 ± 38.3 vs. 7.4 ± 10.0, *P* = 0.018) (Table 1).

**Table 1:** Baseline characteristics of the patients in IVC group and control group

Characteristic parameters	IVC group (*n* = 139)	Control group (*n* = 139)	t / χ^2^	*P*
Age (year)	74.3 ± 7.7	74.4 ± 7.8	0.054	0.957
Female sex- no, (%)	98 (70.5%)	98 (70.5%)	0.000	1.000
Fracture location			–	–
Thoracolumbar region (T11-L2) - no, (%)	139 (100%)	139 (100%)		
Fracture severity			0.000	1.000
Mild	102	102		
Moderate	37	37		
Fracture type			5.067	0.079
Wedge	78	91		
Biconcave	10	14		
Crush	51	34		
Puncture procedure			–	–
Bilateral transpedicular puncture - no, (%)	139 (100%)	139 (100%)		
Cement volume (ml)	5.7 ± 1.8	5.5 ± 1.8	−0.977	0.329
Property of cement			–	–
Low viscosity-no, (%)	139 (100%)	139 (100%)		
Duration of fracture	15.4 ± 38.3	7.4 ± 10.0	−2.382	0.018

*IVC* intravertebral cleft

The rate of type-B, type-S, and venous type leakage was significantly lower in the IVC group compared with the control group (20.9% vs. 31.7%, *P* = 0.041; 24.5% vs. 52.5%, *P* = 0.000; 37.4% vs. 67.6%, P = 0.000) (Table 2).

**Table 2:** Comparisons of the rate of type-B, type-S, and venous type leakage between IVC group and control group

Leakage type	IVC group (*n* = 139)	Control group (*n* = 139)	χ^2^	*P*
Type-B-no, (%)	29 (20.9%)	44 (31.7%)	4.180	0.041
Type-S-no, (%)	34 (24.5%)	73 (52.5%)	23.110	0.000
Venous type-no, (%)	52 (37.4%)	94 (67.6%)	25.446	0.000

*IVC* intravertebral cleft

The rate of type-C, and type-D, and cortical type leakage was significantly greater in the IVC group compared with the control group (25.9% vs. 12.2%, *P* = 0.004; 16.5% vs. 6.5%, *P* = 0.009; 40.3% vs. 16.5%, *P* = 0.000) (Table 3).

**Table 3:** Comparisons of the rate of type-C, type-D and cortical type leakage between IVC group and control group

Leakage type	IVC group (*n* = 139)	Control group (*n* = 139)	χ^2^	*P*
Type-C-no, (%)	36 (25.9%)	17 (12.2%)	8.416	0.004
Type-D-no, (%)	23 (16.5%)	9 (6.5%)	6.922	0.009
Cortical type-no, (%)	56 (40.3%)	23 (16.5%)	19.257	0.000

*IVC* intravertebral cleft

The rate of general leakage was not significant different in IVC group compared with the control group (67.6% vs. 76.3%, *P* = 0.109) (Table 4).

**Table 4:** Comparisons of the rate of leakage in general between IVC group and control group

	IVC group (*n* = 139)	Control group (*n* = 139)	χ^2^	*P*
Leakage in general-no, (%)	94 (67.6%)	106 (76.3%)	2.566	0.109

*IVC* intravertebral cleft

## Discussion

According to previous studies, the impact of IVC on cement leakage in PVP for OVCFs might have been misleading [5–9, 13–15, 18–21]. One of the reasons may be that using too many types of leakage in analysis would make the real impact of IVC underestimated. Considering the leakage mechanism, type-B, type-S as well as a combination type could be deemed the same in nature [30], as there are numerous connections with basivertebral vein and segmental vein, and vertebral venous system is a valveless venous network where blood can flow in either direction [31]. Likewise, type-C, type-D as well as a combination type could be regarded as the same, as all leaks occur through cortical disruption whether at the wall of the vertebral body or/and at the endplates [30]. Therefore, analysis regarding the impact of IVC on two specific leakage types, leakage through vein and leakage though cortex, was carried out in the present study. The result revealed that IVC had a paradoxical impact on occurrence of leakage through vein and cortex, however, it did not significantly influence the incidence of general leakage.

The current study demonstrated that IVC decreased the risk of leakage though vein. One reason may stem from its characteristics of vertebral venous system. In a fractured vertebra with IVC, vertebral venous system might be more vulnerable to secondary occlusion due to the pathophysiological avascularity process, in which the corresponding segmental arteries of the vertebral body would be embolized by thrombosis or damaged by fracture fragments [2], nutritional arteries to the vertebra may be damaged by tiny fractures [32], and medullary arterioles in the vertebra may be disrupted as well [33]. Also, the vertebral venous system would be directly destroyed by progressive collapse of vertebra. On the contrary, in a fractured vertebra without IVC, an active remodeling process, including bone fracture healing process and new vessel formation, restores the continuity of bony structure and revascularization at fracture sites [2], in which contains a richer internal vasculature that intercommunicates with vertebral venous system. Therefore, cement leakage through the venous system may occur less common in the former compared to that in the latter. Another reason may be due to its internal irregularity of cancellous bone. The IVC in the vertebral body usually represents a necrotic cavity, which is similar to the vacuum created during balloon kyphoplasty, thus decreasing the injection forces [18]. The presence of IVC promotes a more controlled filling of the fractured vertebra, decreasing the risk of venous leakage [28]. In addition, IVC resembles a pseudarthrosis surrounded by a fibrocartilaginous membrane or fibrous stroma, partly blocking the path for venous leakage [2, 15, 34]. Clinically, it was supported by most previous investigators who had demonstrated that IVC decreased the rate of leakage through vein, e. g., type-B, type-S, or a combination type [7, 13, 28, 30]. However, Nieuwenhuijse et al. [6]. and Ding et al. [9]. thought that IVC had no significant effect on the occurrence of type-B and type-S. Cement used in the studies was both low-viscosity and medium-viscosity in properties. It was presumed this may bring bias in the mixed series. Wang et al. [18] thought that IVC increased the rate of type-B as a communication between IVC and basivertebral vein was observed on preoperative MRI. Whereas, anatomically or morphologically, existence of the communication was still unknown.

The current study revealed that IVC increased the risk of leakage though cortex. The main reason may be due to its characteristics of cortical bone. An IVC usually represents avascular nonunion, pseudarthrosis, and progressive collapse [1–3]. Pathologic motion or intravertebral instability, in turn, prevents union of fracture fragments [4]. Hence, it was reasonable to postulate that IVC would be significantly correlated with cortical nonunion or defect at the wall or at the endplates. We indeed had noted that cortical defect in IVC group was significantly more common compared with control group (82.0% vs. 52.5%, χ^2^ = 27.462, *P* = 0.000). Another contributor may be the fact that the continuation of the radiolucent line of IVC into the disk space, as well as communication between IVC and intervertebral gaseous collections, have been detected by previous investigations [3, 35]. Those phenomenon predisposes leakage through endplates as well [3]. Clinically, this finding was in agreement with that of most researchers who believed that IVC increased the rate of leakage through cortex, e. g., type-C, type-D, or a combination type [6–9, 13, 19, 28, 30]. Indeed, to the best of our knowledge, no opposing view existed.

IVC had no significant impact on occurrence of general leakage in the present study. The reason may be its paradoxical impact on leakage through vein and leakage through cortex. The results were in accordance with most series [6, 7, 9, 10, 13, 19, 28], though not all [5, 14, 15]. This was presumed that variation exists between series. Meta-analysis might preclude meaningful results, as both vertebroplasty and kyphoplasty series were included as eligible studies [20, 21].

Of note, theoretically, larger cement volume may increase the risk of any type leakage. Whereas, injection of cement was terminated when any cement leakage was noted in a vertebra regardless of the presence of IVC. Hence, it was not surprising that the cement volume in IVC group and control group (5.7 ± 1.8 vs. 5.5 ± 1.8, *P* = 0.329) was comparable, which enhanced the strengthen of the present study.

In brief, the results in the present study implied the inherent characteristics of an IVC in term of its vertebral venous system as well as the cancellous and cortical bone.

Of note, in the present study, there were no complications associated with leakage, e.g., pulmonary embolism and neurological deficit. The reason may be due to a relatively small sample in this study and a very low rate of complications associated with leakage in this procedure [17].

Several limitations must be acknowledged. Firstly, it was an observational case-control study, potential selection bias could not be avoided. Secondly, duration of fracture cannot be matched between two groups, as it varied greatly among cases in the study, from 1 to 720 days. Duration of fracture as recorded may be primitive, as it is difficult to obtain the real fracture age from the patients who suffered fracture spontaneously without any traumatic events, had a few weeks or months symptom-free period after initial traumatic events, and had memory impairment. Therefore, we were unable to cancel out the effect of duration of fracture on cement leakage. Fortunately, previous researchers suggested that duration of fracture was not significantly associated with cement leakage [6, 9]. Thirdly, we did not include cases of severe fracture, most of which had been not considered as candidates for vertebroplasty. This might have biased our results.

## Conclusions

IVC had an opposite impact on occurrence of leakage through vein and cortex, however, it did not significantly influence the incidence of general leakage. The results might provide an insight into the characteristics of IVC in a fractured vertebra regarding its vertebral venous system as well as the cancellous and cortical bone.

## Data Availability

The datasets used during the current study are available from the corresponding author on reasonable request.

## Abbreviations

IVC: Intravertebral cleft
PVP: Percutaneous vertebroplasty
OVCFs: Osteoporotic vertebral compression fractures
BMD: Bone mineral density
PMMA: Polymethylmethacrylate
CT: Computed tomography
MRI: Magnetic resonance imaging
